# Up-regulation of c-MYC and SIRT1 expression correlates with malignant transformation in the serrated route to colorectal cancer

**DOI:** 10.18632/oncotarget.628

**Published:** 2012-10-03

**Authors:** Lydia Kriegl, Michael Vieth, Thomas Kirchner, Antje Menssen

**Affiliations:** ^1^ Department of Pathology, Ludwig-Maximilians-University (LMU), Munich. Germany; ^2^ Department of Pathology, Klinikum Bayreuth, Bayreuth, Germany

**Keywords:** Serrated route, colorectal cancer, SIRT1, c-MYC, BRAF, KRAS mutation

## Abstract

Approximately 7.5% of all colorectal cancers are considered to originate from the alternative, serrated route. Here, we investigate the expression of the c-MYC oncogene and the SIRT1 protein deacetylase by immunohistochemical staining in subgroups of colorectal serrated lesions that were characterized by different molecular alterations. The expression of c-MYC and SIRT1 correlated with the presence of *KRAS* and *BRAF* mutations and high expression of c-MYC and SIRT1 was strongly associated with higher grades of malignancy. In contrast, in the majority of serrated lesions without *KRAS* or *BRAF* mutations, c-MYC and SIRT1 expression was not found increased. In this group only a subset of mostly high grade intraepithelial neoplasia and carcinoma was characterized by elevated c-MYC and SIRT1 expression. This was associated with nuclear localization of beta-catenin, indicating that Wnt pathway activation may confer transcriptional induction of c-MYC. In summary, we established a link between oncogenic K-Ras and B-Raf, suggesting post-transcriptional regulation of c-MYC through MAPK/ERK1/2 pathway activation, as well as for Wnt signalling to the activation of the c-MYC oncogene, and consequently of SIRT1 in the serrated route. The increasing expressions with higher grades of malignancy suggest crucial functions for c-MYC and SIRT1 in the progression of serrated lesions to colorectal cancer. These functions may include antagonizing of apoptosis and senescence, which are characteristic features of serrated lesions.

## INTRODUCTION

Colorectal cancer is the third most common cancer in the world and is one of the leading causes of cancer-related deaths [1]. Malignant transformation in the classical adenoma – carcinoma sequence is crucially associated with the activation of the Wnt/beta-catenin signaling pathway which leads to the transcriptional induction of the c-MYC oncogene [2].

Besides the classical adenoma – carcinoma sequence, strong evidence for an alternative serrated pathway in the development of colorectal cancer has emerged in recent years [3, 4]. The serrated pathway includes lesions with “saw-tooth” crypt morphology like hyperplastic polyps, sessile serrated adenoma, traditional serrated adenoma and invasive serrated adenocarcinoma [3]. In contrast to the classical adenoma–carcinoma sequence, deregulation of the Wnt/beta-catenin pathway is rarely observed, whereas mutations of *BRAF*, and less frequently *KRAS* have been shown to be the initiating events in the serrated route to colorectal cancer [5-8]. Lesions with BRAF mutations are frequently located in the right colon and are strongly associated with MSI and DNA methylation abnormalities, whereas lesions initiating with KRAS mutations predominantly arise in the left colon and are characterized by microsatellite stability (MSS) or microsatellite instability low (MSI-L) [4, 9, 10]. Besides lesions with mutant KRAS or BRAF, a third subset exists which does not display any known aberrant oncogene activation.

We have shown previously that c-MYC activates the SIRT1 enzyme which critically contributes to suppression of senescence and inhibition of c-MYC-induced apoptosis [11]. The NAD^+^ dependent SIRT1 protein deacetylase inhibits various pro-apoptotic factors, including p53 and is necessary for cancer cell, and as recently shown, for cancer stem cell survival [12]. SIRT1 has therefore been suggested to exert tumor promoting functions in human cancers ([13-17], reviewed in [16, 18]). In colorectal cancer of the classical route SIRT1 is overexpressed [11, 19], which correlates with high c-MYC expression [11].

In order to determine an involvement of c-MYC and SIRT1 in the tumorigenesis of the serrated route to colorectal cancer, we analyzed their expression in a well characterized collection of serrated polyps and carcinomas. We demonstrate that high c-MYC and SIRT1 levels are associated with oncogenic BRAF and KRAS, and with activated Wnt signalling. Furthermore, with higher grades of malignancy and invasiveness the expression of c-MYC and SIRT1 consistently increased, implicating a so far unrecognized role of c-MYC and SIRT1 in the tumorigenesis of the serrated route to colorectal cancer.

## RESULTS

### Classification and clinical data of serrated lesions

The final case collection included 121 lesions. BRAF mutation was found in 73 cases comprising 20 hyperplastic polyps, 24 sessile serrated adenomas without intraepithelial neoplasia, 2 sessile serrated adenomas with low grade intraepithelial neoplasia, 8 sessile serrated adenomas with high grade intraepithelial neoplasia, 10 traditional serrated adenomas with low grade intraepithelial neoplasia, 1 traditional serrated adenoma with high grade intraepithelial neoplasia and 8 invasive serrated adenocarcinomas (table 1). KRAS mutation was present in 22 cases including 1 hyperplastic polyp, 1 sessile serrated adenoma without intraepithelial neoplasia, 2 sessile serrated adenomas with high grade intraepithelial neoplasia, 9 traditional serrated adenomas with low grade intraepithelial neoplasia, 1 traditional serrated adenoma with high grade intraepithelial neoplasia and 8 invasive serrated adenocarcinomas. BRAF and KRAS mutations were mutually exclusive. The 26 cases without BRAF and KRAS mutation were comprised of 1 hyperplastic polyp, 7 sessile serrated adenomas without intraepithelial neoplasia, 2 sessile serrated adenomas with low grade intraepithelial neoplasia, 1 sessile serrated adenomas with high grade intraepithelial neoplasia, 2 traditional serrated adenomas with low grade intraepithelial neoplasia, 4 traditional serrated adenomas with high grade intraepithelial neoplasia and 9 invasive serrated adenocarcinomas. Table 1 gives a summary of the location and age of patients. Taken together, sessile serrated lesions with and without intraepithelial neoplasia were more often located in the right colon (72% right sided versus 26% left sided, table 1) and frequently showed BRAF mutations (72% BRAF mutation versus 6 % KRAS mutation, table 1). Traditional serrated adenomas with low and high grade intraepithelial neoplasia derived from the distal colon (19% right sided versus 67% left sided, table 1) and exhibited considerably more often KRAS mutations than sessile serrated adenomas (41% BRAF mutation versus 37 % KRAS mutation, table 1).

**Table 1:** Distribution of location and mutational status of serrated lesions, and age of patients

Histology	Number of cases (n)	Right sided	Left sided	Location n.a.	BRAF mutation (n)	KRAS mutation (n)	BRAF KRAS wt (n)	Average age (y)	Age range (y)
HP	22	8/22 (36%)	14/22 (64%)	-----	20	1	1	64	47 – 85
SSA	32	22/32 (69%)	10/32 (31%)	-----	24	1	7	63	30 – 89
SSA with LGIEN	4	3/4 (75%)	1/4 (25%)	-----	2	-----	2	62	57 – 69
SSA with HGIEN	11	9/11 (82%)	1/11 (9%)	1/11 (9%)	8	2	1	67	59 – 77
TSA with LGIEN	21	4/21 (19%)	16/21 (76%)	1/21 (5%)	10	9	2	68	46 – 89
TSA with HGIEN	6	1/6 (17%)	2/6 (33%)	3/6 (50%)	1	1	4	78	70 – 84
invasive carcinoma	25	7/25 (28%)	14/25 (56%)	4/25 (16%)	8	8	9	71	49 – 84

HP hyperplastic polyp; SSA sessile serrated adenoma; TSA traditional serrated adenoma; LGIEN low grade intraepithelial neoplasia; HGIEN high grade intraepithelial neoplasia; wt wild type; n.a. not applicable

### c-MYC and SIRT1 expression in normal colon mucosa and serrated lesions

**Figure 1:**
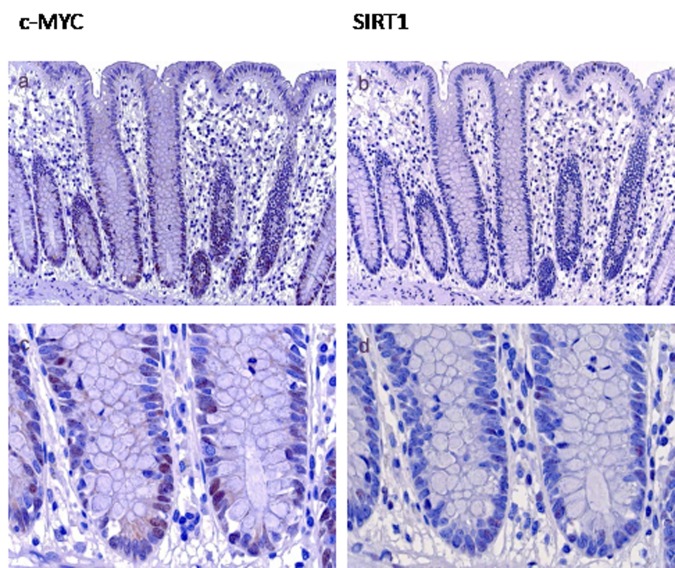
c-MYC and SIRT1 expression in normal mucosa c-MYC expression in normal mucosa was found in the proliferative zone in the basal third of the crypts (Figure 1a, higher magnification in c). Some of these cells were also positive for SIRT1 (b, higher magnification in d) (Original magnification a, b: 100 x, c, d: 630 x)

In normal mucosa a moderate c-MYC expression was restricted to the proliferative zone in the basal third of the crypts (Figure 1a, c). Some of these cells were also positive for SIRT1 (Figure 1b, d). In hyperplastic polyps and sessile serrated adenomas without intraepithelial neoplasia c-MYC expression was mainly localized to one or two thirds of the base of the crypts, corresponding to a low or moderate expression in the majority of cases (51 of 54 cases (94%)) (table 2). SIRT1 was either detected in some scattered cells in the proliferative zone or restricted also to the basal third or two thirds of the crypts (51of 54 cases (95%) (table 2). Irrespective of the molecular alteration and histologic subtype, lesions with low grade intraepithelial neoplasia revealed a moderate to high expression of both, c-MYC (23 of 25 cases (93%)) and of SIRT1 (17 of 25 cases (68%), table 2). Most lesions with high grade intraepithelial neoplasia and invasive carcinomas were characterized by high c-MYC and SIRT1 expression (c-MYC: 76% and 72%; respectively, SIRT1: 64% (both groups), table 2). Taken together, a higher tumor grade was accompanied by a correlating, increasing expression of c-MYC and SIRT1 irrespective of the histologic subtype and side of localization.

**Table 2:** SIRT1 and c-MYC expression in serrated lesions of the colon

Histology	Number of cases (n)	SIRT1 <5%	SIRT1 low	SIRT1 mode-rate	SIRT1 high	c-MYC <5%	c-MYC low	c-MYC mode-rate	c-MYC high
HP/SSA without IEN	54	10/54	22/54	19/54	3/54	1/54	32/54	19/54	2/54
		19%	41%	35%	5%	2%	59%	35%	4%
lesions with LGIEN	25	3/25	5/25	9/25	8/25	-----	2/25	9/25	14/25
		12%	20%	36%	32%		8%	36%	56%
lesions with HGIEN	17	3/17	1/17	2/17	11/17	-----	-----	4/17	13/17
		18%	6%	12%	64%			24%	76%
Invasive Carcinoma	25	5/25	2/25	2/25	16/25	3/25	1/25	3/25	18/25
		20%	8%	8%	64%	12%	4%	12%	72%

HP hyperplastic polyp; SSA sessile serrated adenoma; IEN intraepithelial neoplasia; LGIEN low grade intraepithelial neoplasia; HGIEN high grade intraepithelial neoplasia

### c-MYC and SIRT1 expression with respect to the *BRAF* and *KRAS* mutational status

**Figure 2:**
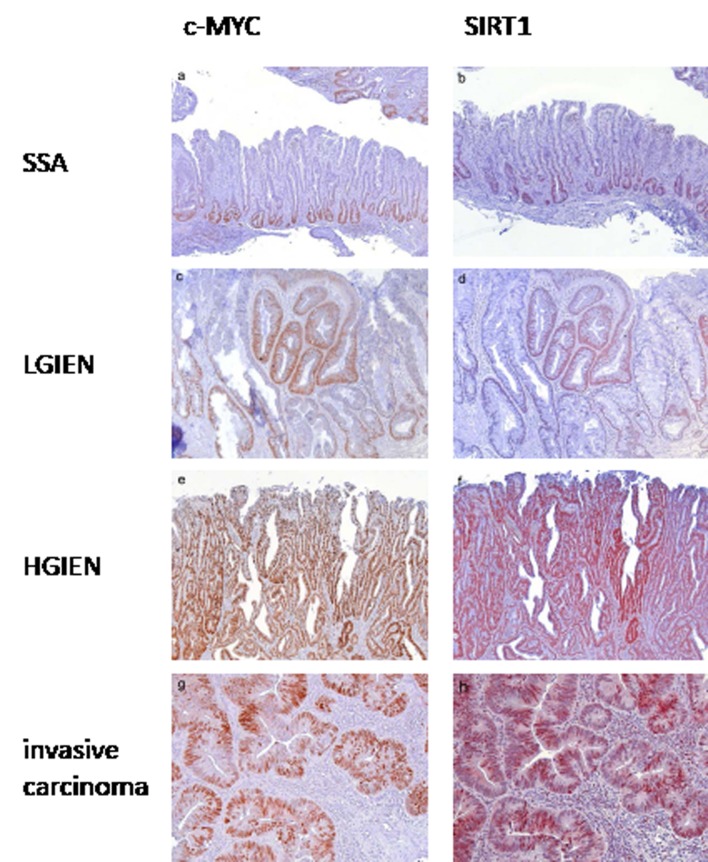
c-MYC and SIRT1 expression in serrated lesions with *BRAF* mutation In sessile serrated adenomas without intraepithelial neoplasia c-MYC (a) and SIRT1 (b) expression was frequently localized to the basal third of the base of the crypts, corresponding to a low expression pattern. In sessile serrated adenomas with low grade intraepithelial neoplasia c-MYC (c) and SIRT1 (d) were frequently found in the complete dypsplastic area corresponding to a strong expression pattern. In sessile serrated adenomas with high grade intraepithelial neoplasia strong expression of c-MYC (e) and SIRT1 (f) was found, which was defined as staining from the base to the surface of the dysplastic crypts. In invasive serrated adenocarcinomas strong expression of c-MYC (g) and SIRT1 (h) was found defined as more than 70% positive tumor cells. Serrated lesions with *KRAS* mutation exhibited a comparable expression pattern. (Original magnification a, b, e, f: 100 x, c, d, g, h: 200 x)

**Figure 3:**
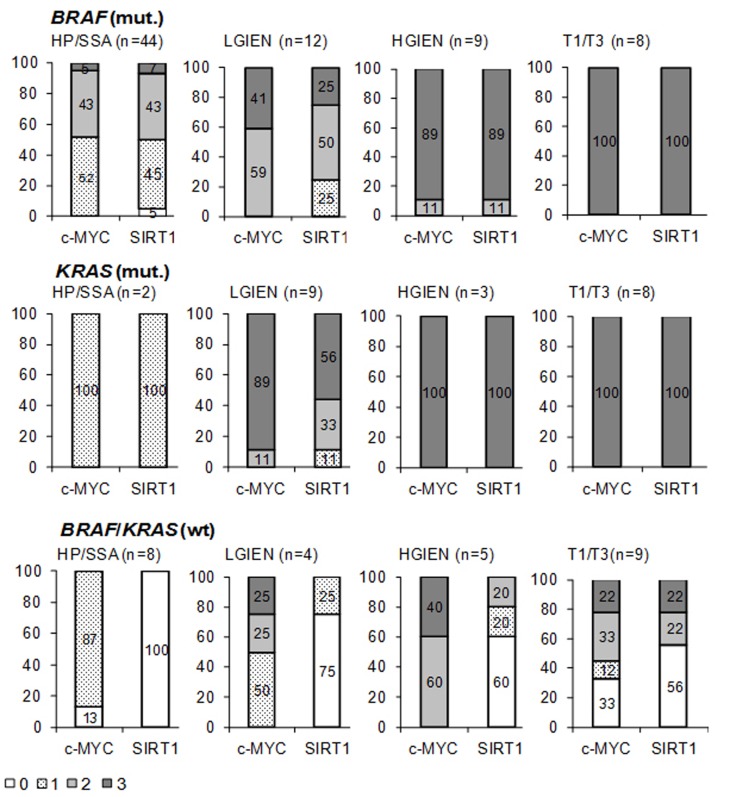
Summary of c-MYC and SIRT1 expression in different groups of serrated lesions and carcinomas expressing mutant or wild type *KRAS* and *BRAF* Lesions exhibiting no detectable (0), low (1), moderate (2) and strong (3) c-MYC and SIRT1 expression are given as the percentage of the total number of cases in the respective group of serrated lesion/carcinomas (see supplementary tables 1-3) HP hyperplastic polyp; SSA sessile serrated adenoma; LGIEN low grade intraepithelial neoplasia; HGIEN high grade intraepithelial neoplasia, T1/3: invasive carcinomas, wt wild type, mut. mutant; n number of cases.

In lesions with BRAF or KRAS mutation c-MYC and SIRT1 expression was low to moderate in hyperplastic polyps and sessile serrated adenomas (Figure 2a, b), moderate to high in lesions with low grade intraepithelial neoplasia (Figure 2c, d), and high in lesions with high grade intraepithelial neoplasia (Figure 2e, f) and invasive serrated carcinomas (Figure 2g, h) (summarized in Figure 3, supplementary tables 1 and 2).

**Figure 4:**
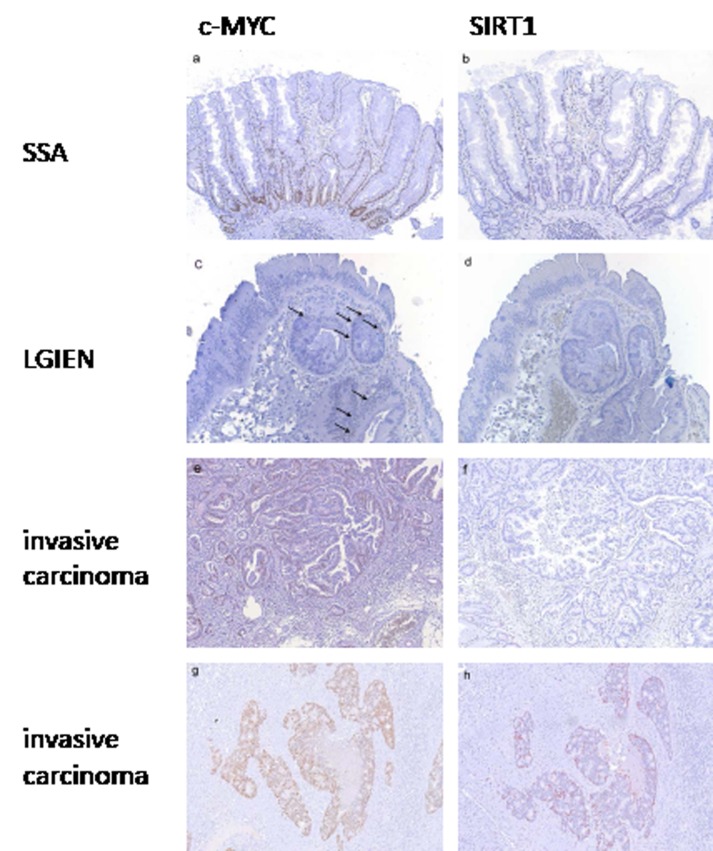
c-MYC and SIRT1 expression in serrated lesions with wild type *BRAF* and *KRAS* In sessile serrated adenomas without intraepithelial neoplasia c-MYC (a) and SIRT1 (b) expression was restricted to the basal third of the crypts corresponding to a low expression pattern. In lesions with low grade intraepithelial neoplasia c-MYC (c) and SIRT1 (d) expression was found in some scattered cells corresponding to a low expression pattern. In invasive carcinomas two groups were found. In one group expression of c-MYC was low (e) with only few positive cells (<30%) and expression of SIRT1 was frequently negative (f). In the other group c-MYC expression was high (g) and SIRT1 expression was at least low to moderate (h). (Original magnification: 100 x)

In contrast, lesions with wild type KRAS and BRAF displayed considerably lower c-MYC and SIRT1 levels. In sessile serrated adenomas and hyperplastic polyps, c-MYC expression was detected in the basal third of the crypts (Figure 4a). In all cases, some scattered cells at the bases of the crypts expressed SIRT1 (Figure 4b). These staining patterns and intensity for c-MYC and SIRT1 were reminiscent of normal crypts. In lesions with low grade intraepithelial neoplasia, c-MYC was expressed at low level (Figure 4c) with very few weakly positive SIRT1 expressing cells distributed throughout the lesions (Figure 4d). In lesions with high grade intraepithelial neoplasia, c-MYC expression was found at a moderate to high level, whereas SIRT1 expression was restricted to single cells. In invasive carcinomas, we identified two groups with respect to the c-MYC and SIRT1 levels: One group comprised four cases (45%) which displayed negative to low c-MYC expression (Figure 4e) and negative SIRT1 staining (Figure 4f). The other group with five cases (55%) showed moderate to high c-MYC expression (Figure 4g) and low to moderate SIRT1 expression (Figure 4h, supplementary table 3). All invasive carcinomas showed similar histologic features with no differences between any of the subgroups. Noteworthy, none of the serrated lesions with wild type BRAF and KRAS displayed an equivalently high c-MYC and SIRT1 expression as the lesions with oncogenic KRAS or BRAF (Figure 3).

### Nuclear beta-catenin expression in serrated lesions

To elucidate whether activation of the Wnt pathway may be involved in c-MYC over-expression, all serrated lesions, with and without KRAS or BRAF mutations were analyzed for nuclear beta-catenin expression (table 3). As documented by the lack of nuclear beta-catenin expression there was no sign of activated Wnt signalling in all sessile serrated adenomas and hyperplastic polyps, except for some cells at the base of the crypts. Nuclear beta-catenin was only found in one sessile serrated adenoma with low grade intraepithelial neoplasia out of 25 (4%) lesions with low grade intraepithelial neoplasia. In lesions with high grade intraepithelial neoplasia nuclear beta-catenin was detected in seven out of 17 cases (41%) comprising three sessile serrated adenomas with high grade intraepithelial neoplasia, and four traditional serrated adenomas with high grade intraepithelial neoplasia. In invasive carcinomas nuclear beta-catenin was found in 14 out of 25 cases (51%), whereby this was not associated with morphological changes. Therefore, nuclear beta-catenin expression does not correlate with any morphological subtype of serrated lesions.

**Table 3:** Correlation of nuclear beta-catenin expression with *KRAS, BRAF* mutation and intraepithelial neoplasia in serrated lesions of the colon

		nuclear localization beta-catenin (number of cases and percent of total)		
	Histology	BRAF mutation	KRAS mutation	KRAS and BRAF wild type
serrated lesions	without IEN	0/44	0/2	0/8
		0%	0%	0%
	with LGIEN	0/12	0/9	1/4
		0%	0%	25%
	with HGIEN	3/9	0/3	4/5
		33%	0%	80%
	invasive carcinoma	3/8	6/8	5/9
		38%	75%	55%
	total	6/73	6/22	10/26
		8%	27%	38%

IEN intraepithelial neoplasia; LGIEN low grade intraepithelial neoplasia; HGIEN high grade intraepithelial neoplasia

### Nuclear beta-catenin in relation to the *BRAF/KRAS* mutational status, c-MYC and SIRT1 expression

Nuclear beta-catenin expression occurred in all three categories of lesions, with increasing prevalence with higher grade of malignancy (table 3). However, the percentage of cases positive for nuclear beta-catenin differed strongly depending on the BRAF/KRAS mutational status: Compared to 38% in the BRAF/KRAS wild type group, and 27% in the mutant KRAS group, nuclear beta-catenin was notably lower in lesions with BRAF mutation (8% (table 3)). Interestingly, high grade intraepithelial lesions with wild type BRAF/KRAS revealed a marked predominance of nuclear beta-catenin (80% versus 27% in the BRAF/KRAS mutant cases).

**Figure 5:**
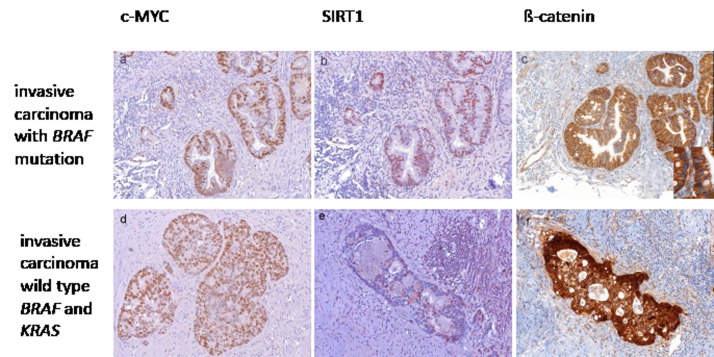
Expression of c-MYC and SIRT1 in correlation with beta-catenin localization High c-MYC (a) and SIRT1 (b) expression in lesions with *BRAF* (shown here) or *KRAS* mutation, in the absence of nuclear beta-catenin staining (c, inset shows higher magnification of negative nuclear staining). In invasive serrated adenocarcinoms with wild type *KRAS* and *BRAF*, c-MYC expression was high (d) and SIRT1 expression was frequently moderate (e) only in those cases which showed nuclear beta-catenin expression (f). (Original magnification a, b, c: 200 x, Inset c 630 x, d, e, f: 100 x)

**Figure 6:**
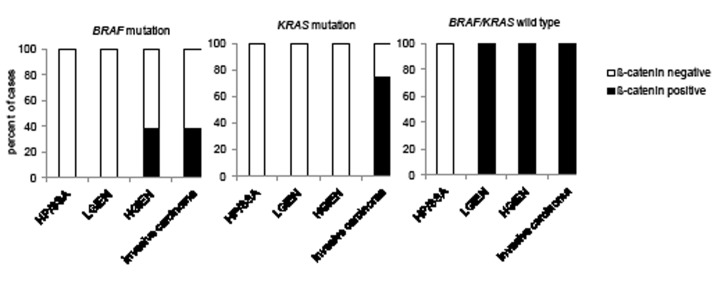
Nuclear beta-catenin expression in *KRAS* and *BRAF* wild type and mutated serrated lesions with high c-MYC expression Percentage of cases with nuclear beta-catenin that exhibit strong (staining: 3) c-MYC expression in wild type and mutant *BRAF* and *KRAS* serrated lesions and invasive carcinomas. HP hyperplastic polyp; SSA sessile serrated adenoma; LGIEN low grade intraepithelial neoplasia; HGIEN high grade intraepithelial neoplasia.

In lesions with BRAF or KRAS mutations, high c-MYC and SIRT1 expression was independent of nuclear beta-catenin expression as the expression level did not differ between beta-catenin positive and negative cases. For instance in some lesions with high grade intraepithelial neoplasia and KRAS mutation that displayed high c-MYC and SIRT1 expression (Figure 5a, b), no nuclear beta-catenin was detected (Figure 5c), whereas other lesions with equally high c-MYC and SIRT1 levels were positive for nuclear beta-catenin (data not shown). In the majority of cases with KRAS or BRAF mutations, high c-MYC expression was consistently observed in lesions with low grade intraepithelial neoplasia to invasive carcinomas (summarized in Figure 3). This did not correlate with nuclear beta-catenin, which was only detected in higher grade lesions (Figure 6). Interestingly, in wild type BRAF and KRAS lesions with high grade intraepithelial neoplasia, moderate to strong c-MYC expression correlated with nuclear beta-catenin localization (Figure 6). In wild type KRAS/BRAF invasive adenocarcinomas with nuclear beta-catenin, increased c-MYC expression was associated with an up-regulation of SIRT1 (Figure 5d, e, f). Conversely, invasive adenocarcinomas negative for nuclear beta-catenin did not reveal any substantial c-MYC and SIRT1 staining (data not shown). Thus, transcriptional activation through the beta-catenin/TCF complex may result in increased c-MYC, and consequently elevated SIRT1 protein levels in serrated lesions without constitutive K-Ras or B-Raf activation. Generally, however the molecular differences between the invasive serrated carcinomas were not associated with any overt morphological alteration.

## DISCUSSION

The evolving concept of serrated lesions of the colon comprises the notion of a sessile and traditional serrated pathway. Sessile serrated adenomas are more frequently found in the proximal colon, initiate through BRAF mutations and are commonly MSI. In contrast, traditional serrated adenomas are more often found in the distal colon, harbour KRAS mutations and are MSI-L or MSS [4, 9, 10]. In our collection the distribution and mutation status of cases reflects this concept. But despite the clear preferences of location and mutation status of serrated lesions, c-MYC and SIRT1 expression was unrelated to the morphologic subtype and side distribution.

A characteristic feature of serrated lesions of the colon is the resistance to apoptosis that has been linked to mutational activation of KRAS and BRAF [7, 24-29]. However, the molecular events downstream of oncogenic KRAS and BRAF responsible for the anti-apoptotic function in serrated lesions have not been completely defined. The pleiotropic SIRT1 deacetylase negatively regulates various pro-apoptotic factors, including p53, thereby antagonizing senescence and apoptosis in cancer cells [13-15, 30, 31] and in cells immortalized by c-MYC [11]. Here, we provide evidence that SIRT1 expression is elevated and correlates with c-MYC in serrated lesions. Furthermore, the expression levels of both proteins increased with higher malignancy of tumors. This is in accordance with recent observations, that c-MYC induces an increase in the level and activity of the NAD^+^-dependent SIRT1 protein deacetylase [11, 32-34] (reviewed in [35]). In this context we have previously identified a positive feedback loop, in which two processes involving the induction of nicotinamide phosphoribosyltransferase (NAMPT) and the sequestration of the endogenous SIRT1 inhibitor protein, deleted in breast cancer 1 (DBC1) mediate the c-MYC-induced activation of SIRT1. Finally, SIRT1 itself potentiates these effects by reducing the rate of degradation of c-MYC [11].

In lesions and invasive carcinomas of the serrated route, high c-MYC and SIRT1 levels were associated with K-Ras, B-Raf, or in a minor fraction, with Wnt pathway activation. Oncogenic mutations of KRAS and BRAF result in constitutive activation of the MAPK/ERK1/2 pathway and c-MYC phosphorylation [36, 37]. Upon phosphorylation by ERK1/2, degradation of c-MYC is inhibited through the K-Ras-induced activation of PI3K [38, 39]. Thus, the association with mutant KRAS and BRAF suggests that the up-regulation of c-MYC may be mediated by protein stabilization through MAPK/ERK1/2 pathway activation. In a smaller group of more advanced lesions without these mutations, c-MYC may be transcriptionally induced by the beta-catenin/TCF complex [2], since high levels of c-MYC were associated with the nuclear localization of beta-catenin. Aberrant nuclear accumulation of beta-catenin has been reported previously for serrated lesions [40-42]. Whereas mutations of the APC gene are very rare in serrated lesions, and mutations in the gene encoding for beta-catenin have not been observed so far [41, 43], hypermethylation of the APC promoter has been described for a subset of serrated adenomas [43]. Intriguingly, nuclear beta-catenin displayed the highest prevalence in wild type KRAS and BRAF lesions. Therefore, our data suggest, that enabling c-MYC activation either through oncogenic K-Ras or B-Raf, or transcriptionally by deregulation of Wnt signalling is crucial in both, the serrated route as well as in the classical route to colorectal cancer [2].

Higher levels of c-MYC can induce activation of p53 and apoptosis [44, 45]. However, as we have shown before, the c-MYC-mediated SIRT1 activation interferes therewith, since it suppresses p53-dependent and -independent apoptosis and antagonized senescence [11]. Hence, unscheduled c-MYC expression together with elevated SIRT1 activity in serrated lesions and invasive carcinomas may repress the two major tumor suppressive mechanisms, apoptosis and senescence and thus support cell survival, expansion and cancer progression. In line with such a role of SIRT1, only 17% of colorectal carcinomas with BRAF mutations and MSI, which are believed to originate from serrated precursor lesions, display p53 mutations [46]. Our previous studies of human samples and of a mouse model have revealed that serrated polyps and adenomas display high expression of the CDK inhibitor p16INK4a, a characteristic mark for senescence, which is lost in invasive carcinomas by CDKN2A promoter hypermethylation [7, 8, 47, 48]. SIRT1 localizes to promoters of aberrantly methylated genes [49] and it is weakly expressed in senescent, but high in immortalized cells [50]. In serrated lesions SIRT1 may therefore also be involved in antagonizing oncogene-induced senescence. This may be mediated through effects of SIRT1 on DNA methyltransferase 1 (DNMT1) activity, a key enzyme in DNA methylation [51]. Deacetylation of DNMT1 at specific lysines by SIRT1 enhances its methyltransferase activity. In line with these findings, increased SIRT1 expression was found significantly associated with CpG island methylator phenotype (CIMP)-high, MSI-high phenotype, and a high tumor grade in colorectal carcinoma [19]. Future studies will reveal the relevance of the effects in this novel crosstalk of the epigenetic regulators DNMT1 and the histone deacetylase SIRT1 in different cellular contexts and especially in serrated lesions of the colon. Interestingly, recent studies revealed that in chronic myelogenous leukemia (CML) SIRT1 inhibition can prevent the acquisition of mutations and may therefore represent a treatment to overcome drug resistance [52]. In addition, in a BCR-ABL transgenic mouse model inhibition of SIRT1 in combination with the tyrosine kinase inhibitor Imatinib leads to p53 activation-mediated elimination of cancer stem cells, indicating that SIRT1 inhibition may represent a strategy for targeting cancer stem cells [12].

Taken together, we provide evidence that c-MYC and SIRT1 are crucially involved in the alternative, serrated pathway to colorectal cancer. Thereby, oncogenic functions of c-MYC and properties of SIRT1, such as antagonizing apoptosis and senescence and/or epigentic regulation may contribute to tumorigenesis and may represent a novel target for future therapies. However, further studies will have to elucidate the exact functional role of SIRT1 in serrated lesions and determine the signalling pathways involved in the development of lesions without oncogenic BRAF or KRAS mutations.

## METHODS

### Specimens

Patient material was taken from the archives of the Department of Pathology, Ludwig-Maximillians-University, Munich. In total, formalin-fixed paraffin-embedded tissue from 121 serrated lesions was obtained. Two independent observers (T.K. and L.K.) classified all samples by applying the criteria of Torlakovic et al. [9]. Our study enrolled hyperplastic polyps, sessile serrated adenomas without intraepithelial neoplasia, sessile serrated adenomas with low grade intraepithelial neoplasia, traditional serrated adenomas with low grade intraepithelial neoplasia, traditional serrated adenomas and sessile serrated adenomas with high grade intraepithelial neoplasia and invasive serrated adenocarcinomas (Table 1).

All cases were classified with regard to BRAF and KRAS mutational status. KRAS was mutated in 22 (18%) cases, and BRAF mutations were detected in 73 (60%) cases, respectively. In 26 (22%) cases, neither KRAS nor BRAF was mutated.

### Immunohistochemistry

Immunohistochemical staining was done on 5 μm tissue sections of FFPE tumor samples. For SIRT1 and c-MYC, consecutive tissue sections were used for immunohistochemistry. SIRT1 monoclonal rabbit antibody (Epitomics, CA, USA, dilution 1: 80, Cat. No. 1104-1), c-MYC monoclonal rabbit antibody (Epitomics, CA, USA, dilution 1: 150, Cat. No. 1472-1), and beta-catenin monoclonal mouse antibody (BD Biosciences, NJ, USA, dilution 1:300, Cat. No. 610154) were used as primary antibodies. For detection of SIRT1 and c-MYC, sections were pre-treated for antigen retrieval by boiling in a microwave oven, twice for 15 min at 750 W in Target Unmasking Fluid (Pan Path, Budel, Netherlands). For the beta-catenin staining, antigen retrieval was done by pre-treatment in ProTaqs IV Antigen-Enhancer (Quartett, Berlin, Germany). Endogenous peroxidase was blocked using 7.5% hydrogen peroxide for 10 minutes. Vectastain ABC-Kit Elite Universal (Vector Laboratories, CA, USA) was used for beta-catenin detection. AEC*+* high sensitivity substrate chromogen (Dako, Glostrup, Denmark) was used as a chromogen for SIRT1, DAB*+* substrate chromogen system (Dako, Glostrup, Denmark) for c-MYC and beta-catenin. All slides were counterstained with Hematoxylin (Vector Laboratories, CA, USA). To confirm staining specificity, system controls without primary antibodies, as well as immunoglobulin isotype control antibodies were included.

### Evaluation of c-MYC, SIRT1 and beta-catenin staining

c-MYC and SIRT1 expression were evaluated regarding nuclear expression pattern ranging from 0 – 3, for negative, low, moderate and strong staining. In hyperplastic polyps and sessile serrated adenomas, positive staining of the basal third of the crypts was defined as low. Moderate was defined as staining of the basal two thirds of the crypts, and strong was defined as staining from the base to the surface of the crypts. In sessile serrated adenomas with low grade and high grade intraepithelial neoplasia, only areas with intraepithelial neoplasia were taken into account. In traditional serrated adenomas and invasive adenocarcinomas, the percentage of positive cells was evaluated including negative (0%), low (1-30%), moderate (31-70%) and strong (71-100%) expression.

Regarding beta-catenin staining, only nuclear beta-catenin expression was taken into account. beta-catenin, c-MYC and SIRT1 staining that was confined to some nuclei of scattered cells at the bases of crypts (<5% positive cells) was considered as a normal mucosa staining pattern (Figure 1), which is consistent with the proliferative zone due to physiologically active Wnt signalling within the progenitor population of intestinal epithelium [20].

### Analyses of *KRAS-/ BRAF* mutations

For the analyses of KRAS exon 2 codon 12/13 and BRAF(V600E) exon 15 mutations, genomic DNA was extracted from microdissected serrated lesions, as previously described for KRAS [21]. Pyro-sequencing was done using the Pyro-Gold kit (Qiagen, Germany) and HotStar Taq-Polymerase (Qiagen, Germany). The PF2 primer was used to determine anti-sense sequences. The PyroMark Q24 device (Qiagen, Germany) and the PyroMark™ Q24 software were used for sequencing, and sequence analyses [22, 23].

## Supplementary Tables


