# Supplementary material for: Up-regulation of c-MYC and SIRT1 expression correlates with malignant transformation in the serrated route to colorectal cancer
Source: Oncotarget. 2012 Oct 3;3(10):1182–93. doi: 10.18632/oncotarget.628 (PMC3717960; doi:10.18632/oncotarget.628)
Supplement: Supplementary file 1 [file oncotarget-03-1182-s001.doc]

**Up-regulation of c-MYC and SIRT1 expression correlates with malignant transformation in the serrated route to colorectal cancer –** Kriegl et al

| **Supplementary Table 1: SIRT1 and c-MYC expression in serrated lesions of the colon with *BRAF* mutation** | | | | | | | | | |
| --- | --- | --- | --- | --- | --- | --- | --- | --- | --- |
| Histology | Number of cases  (n) | SIRT1 <5% | SIRT1 low | SIRT1 moderate | SIRT1 high | c-MYC low | c-MYC moderate | c-MYC high |  |
|  |  |  |  |  |  |  |  |  |  |
|  |  |  |  |  |  |  |  |  |  |
| HP | 20 | 1/20 | 8/20 | 8/20 | 3/20 | 9/20 | 9/20 | 2/20 |  |
|  |  | 5% | 40% | 40% | 15% | 45% | 45% | 10% |  |
| SSA | 24 | 1/24 | 12/24 | 11/24 | ----- | 14/24 | 10/24 | ----- |  |
|  |  | 4% | 50% | 46% |  | 58% | 42% |  |  |
| SSA with LGIEN | 2 | ----- | ----- | 2/2 | ----- | ----- | 2/2 | ----- |  |
|  |  |  |  | 100% |  |  | 100% |  |  |
| SSA with HGIEN | 8 | ----- | ----- | 1/8 | 7/8 | ----- | 1/8 | 7/8 |  |
|  |  |  |  | 12% | 88% |  | 12% | 88% |  |
| TSA with LGIEN | 10 | ----- | 3/10 | 4/10 | 3/10 | ----- | 5/10 | 5/10 |  |
|  |  |  | 30% | 40% | 30% |  | 50% | 50% |  |
| TSA with HGIEN | 1 | ----- | ----- | ----- | 1/1 | ----- | ----- | 1/1 |  |
|  |  |  |  |  | 100% |  |  | 100% |  |
| Invasive Carcinoma | 8 | ----- | ----- | ----- | 8/8 | ----- | ----- | 8/8 |  |
|  |  |  |  |  | 100% |  |  | 100% |  |
| HP hyperplastic polyp; SSA sessile serrated adenoma; TSA traditional serrated adenoma; | | | | | | | | |  |
| LGIEN low grade intraepithelial neoplasia; HGIEN high grade intraepithelial neoplasia | | | | | | | |  |  |

|  | **Supplementary Table 2: SIRT1 and c-MYC expression in serrated lesions of the colon with *KRAS* mutation** | | | | | | | | | | | | | | | | |  | | | |
| --- | --- | --- | --- | --- | --- | --- | --- | --- | --- | --- | --- | --- | --- | --- | --- | --- | --- | --- | --- | --- | --- |
|  | | Histology | | Number of cases  (n) | | SIRT1 low | | SIRT1 moderate | | SIRT1 high | | c-MYC low | | c-MYC moderate | | c-MYC high | | |  | | |
|  |  |  |  |  |  |  |  |  |  |  |  |  |  |  |  |  |  |  |  |  |  |
|  | | HP | | 1 | | 1/1 | |  | | ----- | | 1/1 | | ----- | | ----- | | |  | | |
|  | |  |  |  |  | 100% | |  |  |  |  | 100% | |  |  |  |  |  |  | | |
|  | | SSA | | 1 | | 1/1 | | ----- | | ----- | | 1/1 | | ----- | | ----- | | |  | | |
|  | |  |  |  |  | 100% | |  |  |  |  | 100% | |  |  |  |  |  |  | | |
|  | | SSA with HGIEN | | 1 | | ----- | | ----- | | 1/1 | | ----- | | ----- | | 1/1 | | |  | | |
|  | |  |  |  |  |  |  |  |  | 100% | |  |  |  |  | 100% | | |  | | |
|  | | TSA with LGIEN | | 9 | | 1/9 | | 3/9 | | 5/9 | | ----- | | 1/9 | | 8/9 | | |  | | |
|  | |  |  |  |  | 11% | | 33% | | 56% | |  |  | 11% | | 89% | | |  | | |
|  | | TSA with HGIEN | | 2 | | ----- | | ----- | | 2/2 | | ----- | | ----- | | 2/2 | | |  | | |
|  | |  |  |  |  |  |  |  |  | 100% | |  |  |  |  | 100% | | |  | | |
|  | | Invasive Carcinoma | | 8 | | ----- | | ----- | | 8/8 | | ----- | | ----- | | 8/8 | | |  | | |
|  | |  |  |  |  |  |  |  |  | 100% | |  |  |  |  | 100% | | |  | | |
|  | | HP hyperplastic polyp; SSA sessile serrated adenoma; TSA traditional serrated adenoma; | | | | | | | | | | | | | | | | |  | | |
|  | | LGIEN low grade intraepithelial neoplasia; HGIEN high grade intraepithelial neoplasia | | | | | | | | | | | | | | | | |  | | |
|  | | **Supplementary Table 3: SIRT1 and c-MYC expression in serrated lesions of the colon without KRAS or BRAF mutation (WT)** | | | | | | | | | | | | | | | | |  | | |
| Histology | | | Number of cases  (n) | | SIRT1 <5% | | SIRT1 low | | SIRT1 moderate | | SIRT1 high | | c-MYC <5% | | c-MYC low | | c-MYC moderate | | | c-MYC high |  |
|  |  |  |  |  |  |  |  |  |  |  |  |  |  |  |  |  |  |  |  |  |  |
|  |  |  |  |  |  |  |  |  |  |  |  |  |  |  |  |  |  |  |  |  |  |
| HP | | | 1 | | 1/1 | | ----- | | ----- | | ----- | | 1/1 | | ----- | | ----- | | | ----- |  |
|  |  |  |  |  | 100% | |  |  |  |  |  |  | 100% | |  |  |  |  |  |  |  |
| SSA | | | 7 | | 7/7 | | ----- | | ----- | | ----- | | ----- | | 7/7 | | ----- | | | ----- |  |
|  |  |  |  |  | 100% | |  |  |  |  |  |  |  |  | 100% | |  |  |  |  |  |
| SSA with LGIEN | | | 2 | | 1/2 | | 1*/2 | | ----- | | ----- | | ----- | | 1/2 | | 1*/2 | | | ----- |  |
|  |  |  |  |  | 50% | | 50% | |  |  |  |  |  |  | 50% | | 50% | | |  |  |
| SSA with HGIEN | | | 1 | | 1/1 | | ----- | | ----- | | ----- | |  | | ----- | | 1/1 | | | ----- |  |
|  |  |  |  |  | 100% | |  |  |  |  |  |  |  | |  |  | 100% | | |  |  |
| TSA with LGIEN | | | 2 | | 2/2 | | ----- | | ----- | | ----- | | ----- | | 1/2 | | 1/2 | | | ----- |  |
|  |  |  |  |  | 100% | |  |  |  |  |  |  |  |  | 50% | | 50% | | |  |  |
| TSA with HGIEN | | | 4 | | 2*/4 | | 1*/4 | | 1*/4 | | ----- | | ----- | | ----- | | 1*/4 | | | 3*/4 |  |
|  |  |  |  |  | 50% | | 25% | | 25% | |  |  |  |  |  |  | 25% | | | 75% |  |
| Invasive Carcinoma | | | 9 | | 5/9 | | 2*/9 | | 2*/9 | | ----- | | 3/5 | | 1/9 | | 3*/9 | | | 2*/9 |  |
|  |  |  |  |  | 56% | | 22% | | 22% | |  |  | 33% | | 12% | | 33% | | | 22% |  |
| HP hyperplastic polyp; SSA sessile serrated adenoma; TSA traditional serrated adenoma; | | | | | | | | | | | | | | | | | | | |  |  |
| LGIEN low grade intraepithelial neoplasia; HGIEN high grade intraepithelial neoplasia | | | | | | | | | | | | | | | | |  | | |  |  |
| * cases with nuclear beta-catenin expression | | | | | | | | |  | |  | |  | |  | |  | | |  |  |
